# The usefulness of the narrow band imaging (NBI) in decision-making process regarding second look procedure (SL) in laryngeal cancer follow-up after transoral laser microsurgery

**DOI:** 10.1371/journal.pone.0236623

**Published:** 2020-08-07

**Authors:** Joanna Witkiewicz, Hanna Klimza, Krzysztof Piersiala, Joanna Jackowska, Małgorzata Wierzbicka

**Affiliations:** 1 Department of Otolaryngology, Head and Neck Surgery, Poznań University of Medical Sciences, Poznań, Poland; 2 Division of ENT Diseases, Department of Clinical Sciences, Intervention and Technology, Karolinska Institutet, Stockholm, Sweden; 3 Department of Otorhinolaryngology, Karolinska University Hospital, Stockholm, Sweden; 4 Institute of Human Genetics, Polish Academy of Sciences, Poznań, Poland; University of California, Davis, UNITED STATES

## Abstract

**Background:**

The prognostic value of positive surgical margins after transoral laser microsurgery (TOLM) is still under debate. In case of positive superficial margins, some experts recommend a second-look surgery (SL) and some recommend wait and watch approach with close observation. Narrow band imaging (NBI) is an advanced imaging system used to enhance visualization of mucosal vascular pattern. In laryngology, NBI is used to improve the detection of premalignant, dysplastic and malignant lesions.

**Aim:**

To assess the usefulness of NBI imaging in guiding clinical decision making regarding follow-up plan and SL after TOLM.

**Materials and methods:**

A prospective cohort of 127 patients was divided into three groups based on the histology results and NBI vascular pattern of the mucosa. Group A (24/127, 18.90%) consisted of patients with suspicious vascular pattern in NBI or/and with positive deep margin. Group B (52/127, 40.94%) consisted of patients with positive or uncertain superficial margins and non-suspicious vascular pattern in NBI. Group C (51/127, 40.15%) had non-suspicious vascular NBI pattern and all negative margins.

**Results:**

After the first TOLM procedure, 9/24 (37.5%) patients had positive deep margins, 1/24 (4.2%) had uncertain deep margin and 1/24 patient (4.2%) had both positive deep margin and suspicious vascular pattern in NBI. The remaining 13 cases in Group A had a suspicious NBI finding only during the first follow-up. All of the 24 patients (Group A) underwent a second look surgery. The final histology after SL showed squamous cell carcinoma in 10/24 (41.7%) patients. All 10 patients had suspicious vascular pattern in NBI and one patient had both a positive deep margins after the first TOLM and positive NBI finding. None of Group B and C subjects developed an early recurrence.

**Conclusions:**

Our study provides evidence that NBI imaging will be a useful adjunct to margin status after TOLM and will facilitate clinical decision-making regarding performing the SL in patients with positive or uncertain superficial surgical margins in the first TOLM procedure. However, additional investigation with more subjects is required at this time to further validate this technique and change the standard of care.

## Introduction

Transoral laser microsurgery (TOLM) is an effective treatment modality for patients with early glottic cancer. Recently, it has been used with a success in treatment of moderately advanced glottic tumours [1]. A good intraoperative visualization of the tumour, careful delineation of the margins and a close post-treatment surveillance are key determinants of a successful TOLM.

Sun et al. in a recent meta-analysis showed that narrow band imaging (NBI) is a useful technique characterised by high diagnostic value and clinical practicability for the diagnosis of laryngeal cancer [2]. NBI is a method visualizing the vascular pattern of the mucosa with narrow-band light wavelengths (415 and 540 nm). It facilitates the detection of the pathological intraepithelial, papillary capillary loops. The aforementioned findings in NBI are identified as well-demarcated areas with scattered brownish spots [3]. It has been proven that narrow band imaging (NBI) is a useful tool in the detection of early glottic cancer and in the intraoperative delineation of superficial margins during cordectomy [4–6]. It has also been showed that NBI guides clinical decision-making regarding treatment of patients with laryngeal leukoplakia [7,8]. In 2016, European Laryngeal Society (ELS) proposed a new classification for glottic lesions visualised in NBI, which we used to assess the laryngeal mucosa vascular pattern in this study. Premalignant and malignant glottic lesions are characterized by perpendicular vessels in the third dimension towards the surface of the epithelium and are defined as “positive NBI lesions” [9]. The aim of this study was to assess the usefulness of NBI imaging in guiding clinical decision making regarding a follow-up plan and SL after TOLM. In particular, we wanted to address the question, whether data obtained from NBI combined with margin status help to determine the appropriate follow-up scheme in patients after TOLM.

## Material and methods

### Ethical approval

All procedures performed in studies involving human participants were in accordance with the ethical standards of the institutional and national research committee and with the 1964 Helsinki declaration and its later amendments or comparable ethical standards. The study protocol was approved by Bioethics Committee of Poznan University of Medical Sciences. Informed written consent was obtained from all individual participants included in the study.

### Study protocol

This is a prospective study designed to validate the diagnostic accuracy of NBI in post-TOLM laryngeal mucosa evaluation. 127 consecutive patients were included in the study. The inclusion criteria were: (1) diagnosis of laryngeal squamous cell carcinoma, (2) T1-T3N0M0 in TNM staging system, (3) willingness to participate, (4) TOLM procedure performed between 2016 and 2017 in Poznan University of Medical Science, Department of Otolaryngology, Poznan, Poland. The exclusion criteria were: (1) nodal involvement and/or distant metastases, (2) tumour classified as T4 in TNM staging system.

**Fig 1:**
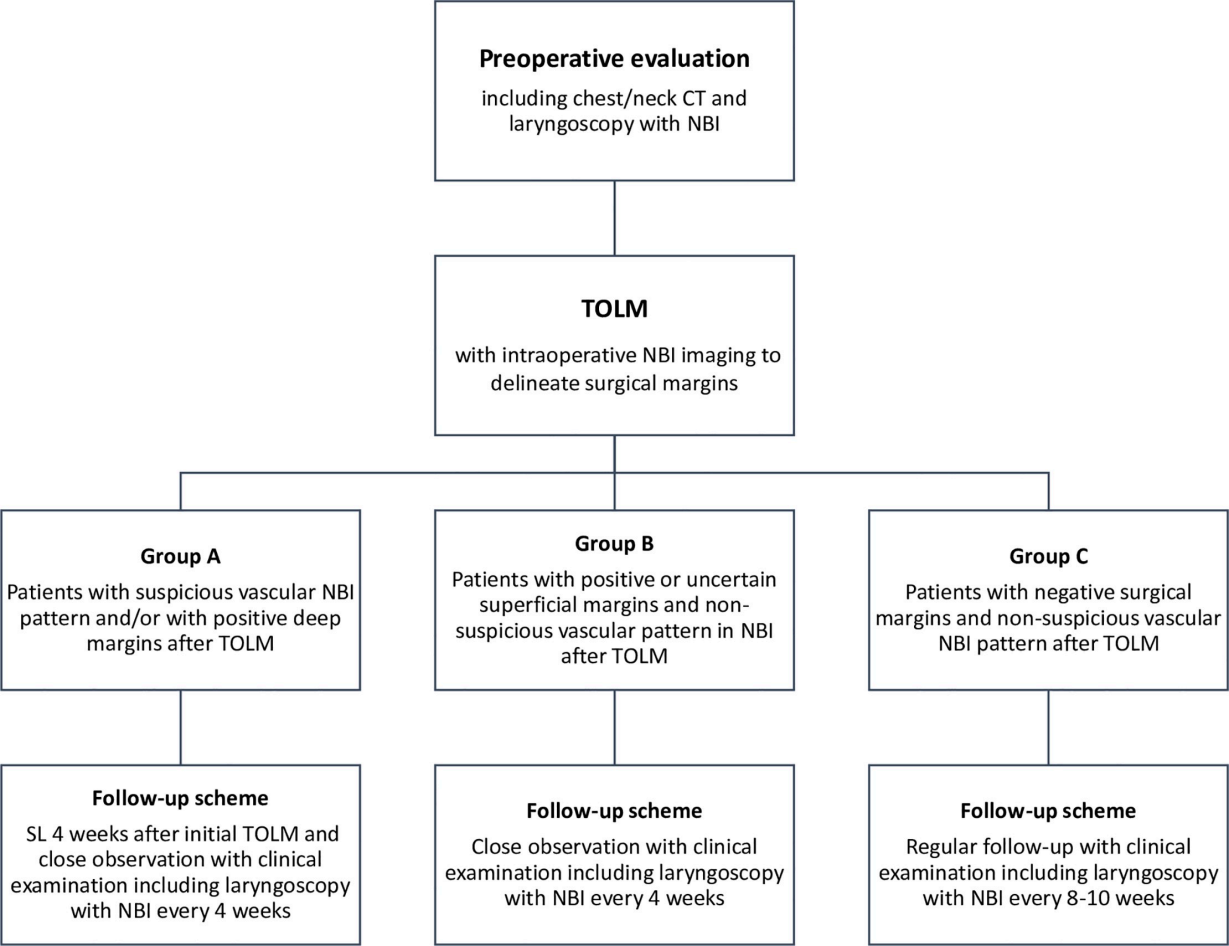
Study protocol. Abbreviations: CT–computed tomography, NBI–narrow band imaging, TOLM–transoral laser microsurgery.

All subjects underwent a preoperative evaluation for extent of disease including clinical examination with flexible laryngoscopy with NBI filter and a chest/neck CT. The NBI was also used intraoperatively to delineate margins. All patients meeting inclusion criteria who underwent TOLM were stratified into three follow-up groups according to the histological margins status and the NBI results during the first follow-up visit as described below. Based on margins histology and NBI, we distinguished Group A with patients who required SL surgery combined with excisional biopsy. These patients had suspicious vascular pattern in NBI visualised during first follow-up visit and/or positive deep margin (R1d) after the initial TOLM. In all patients classified to Group A, excisional biopsy with a cold instrument under general anaesthesia was performed 4 weeks after the first procedure. Group B included patients with positive or uncertain superficial margins after TOLM but not having a suspicious NBI vascular pattern visualised during the first follow-up visit. Those patients were followed up every 4 weeks with close observation. Group C required standard follow-up (every 8–10 weeks) and consisted of those patients who had non-suspicious vascular NBI pattern during the first follow up visit and had all negative margins after TOLM. The assignment of the participants into Group A, Group B and Group C was co-ordinated by experienced head and neck surgeons (MW, JJ, HK), who have at least six years of experience in use of NBI method to assess laryngeal mucosa. Both watchful waiting and SL are considered a standard-of-care in management of laryngeal cancer. Characteristics of the groups are summarised in Table 1 and presented in study flow chart (Fig 1). Patients who denied participation in the study underwent SL always in case of margins positivity disrespecting NBI findings.

**Table 1:** Summary of the results.

Group	Definition of the group	Margins status in TOLM / NBI pattern in follow-up / Histology after SL	Outcome–recurrence
A	SL group: Patients with suspicious vascular pattern in NBI and/or with positive deep margins after TOLM	9 positive deep margins / 1 suspicious vascular NBI pattern / 0 proved ca in SL	1 (4%) patient with both positive deep margins and suspicious pattern in NBI developed local recurrence
		2 close deep margin / 0 suspicious vascular pattern in NBI / 0 proved ca in SL	
		5 negative, 2 uncertain superficial, 6 positive superficial / 13 suspicious vascular NBI pattern / 4 proved ca in SL	
B	Close follow-up: Patients with positive or uncertain superficial margins and non-suspicious vascular pattern in NBI after TOLM	15 positive superficial margins	1 (1.9%) patient with uncertain margin developed local recurrence. He presented with suspicious vascular pattern in NBI in 16^th^ month of follow-up.
		37 uncertain superficial margins	
C	Patients with negative surgical margins and non-suspicious vascular pattern in NBI after TOLM	No risk factors	None of the patients developed recurrence

Abbreviations: SL–second look surgery, TOLM–transoral laser microsurgery, NBI–narrow band imaging.

### Definitions of surgical margins status

Positive margins were defined as those <1 mm, negative >1 mm, and uncertain/close equal to 1 mm.

### Follow-up scheme

**Fig 2:**
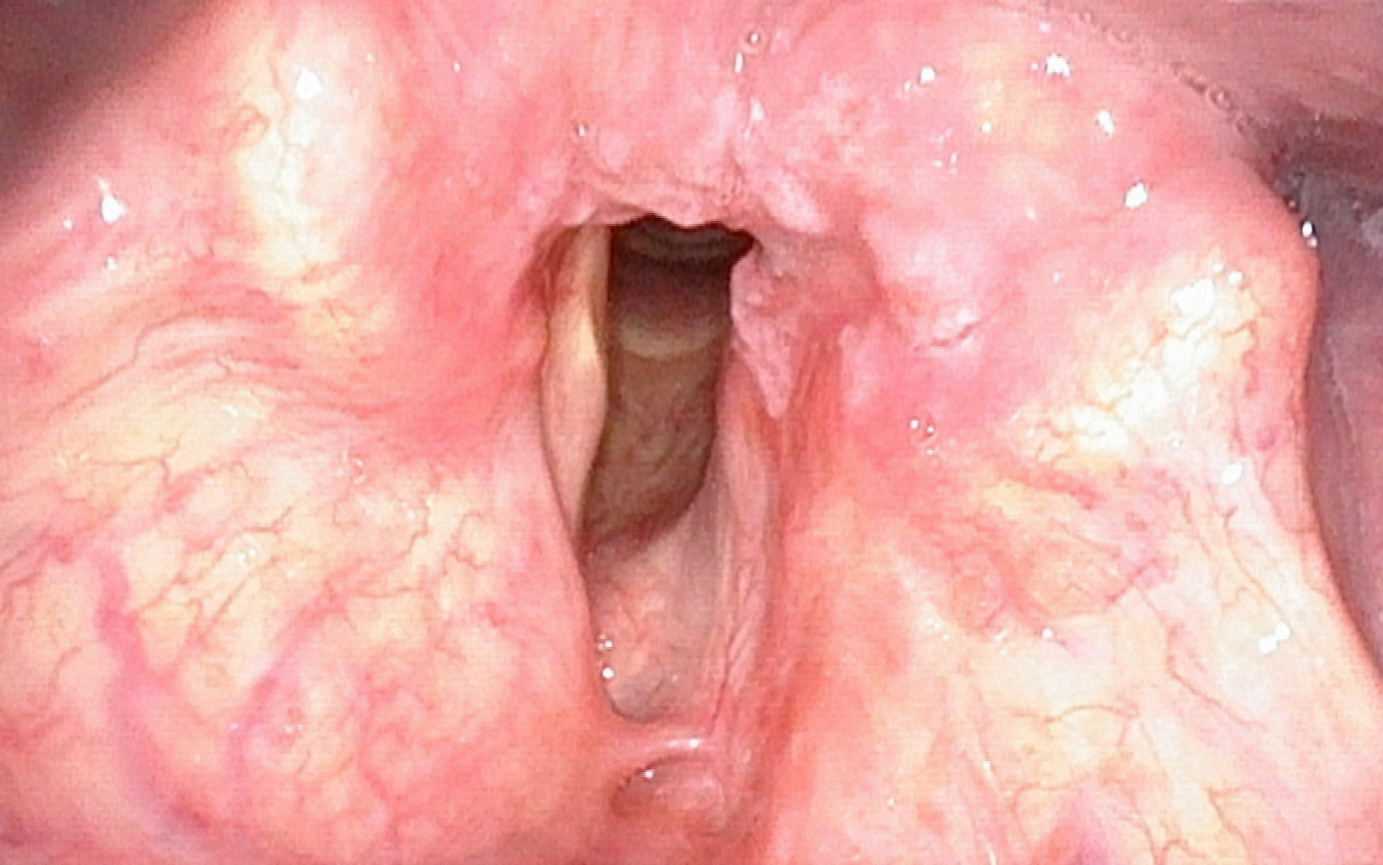
Mucosa assessed in conventional white-light endoscopy.

**Fig 3:**
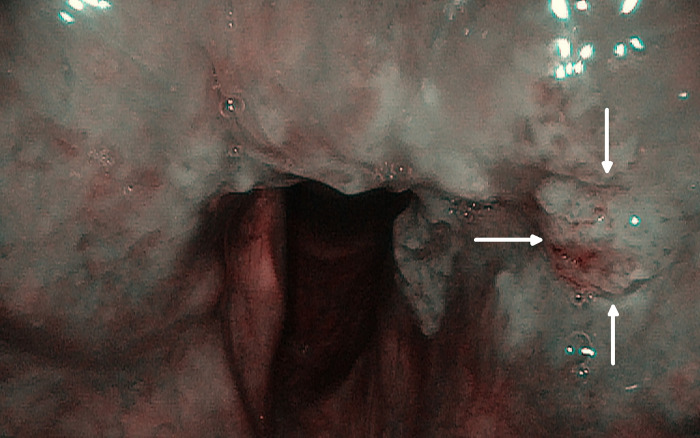
Mucosa with suspicious vascular pattern in NBI. Arrows indicate areas of concern.

All patients were followed for 24–48 months, 36 in average. During the first and every following follow-up visit (every 4 weeks–Group A and B, or 8/10 weeks–Group C) the mucosa of the larynx was assessed by trans-nasal flexible video-endoscope (Olympus Medical System Corporation, Tokyo, Japan) firstly in the white light endoscopy (Fig 2) and afterwards using the optical filter for NBI (Fig 3). The diagnostic criteria defining suspected character of the lesion in NBI endoscopy were as follows: (1) symmetrical arranged brown dot-like loops with narrow-angled turning point, which correspond to perpendicular vascular changes according to European Laryngological Society (ELS) classification [8] and (2) submucosal lesions with amputation of vessels. The patients with aforementioned lesions were qualified to Group A during the first follow-up visit.

The main predictive variables were non-suspicious/suspicious vascular pattern in NBI during the first follow-up visit after TOLM and final histology of SL surgery. The additional variables were age, sex, T stage of tumour, superficial and deep margins status after first TOLM. The primary outcome measure was the correlation between the NBI vascular pattern of mucosa during the first follow-up visit after TOLM and the result of the final histology after the SL.

### Statistical analysis

The statistics were analyzed using the statistical package program Statistica v12 (Statsoft Polska, Krakow, Poland). Chi square test was used to explore relationship between categorical variables and final histology result. The level of significance for all the statistical tests was set at 5% (P < .05). As no previous research was available on which to base the standard deviation estimates and this was an exploratory prospective study, no sample size calculations were made.

## Results

### Patient characteristics

127 consecutive patients with confirmed diagnosis of laryngeal cancer meeting inclusion criteria were included in the study. There were 113 men (88.9%) and 14 women (11.1%). The average age was 63.4 years (range 28 to 90 years). The patients were followed up on average for 23 months (range 13–35 months). Tumour T-stage was as follows: 57 –T1 (44,9%), 57 –T2 (44,9%), 13 –T3 (10,2%). All of the enrolled subjects were cN0 and M0. None of the subjects underwent cervical lymphadenopathy as part of the treatment.

### Group A–A high-risk group

To Group A belonged patients with suspicious vascular pattern in NBI (Fig 3) and/or with positive deep margins after TOLM as described in Methods. 24/127 (18.90%) subjects met inclusion criteria and were assigned to this group. There were 22 (91.67%) males, 2 (8,33%) females and the mean age was 61 years. The patients were classified regarding tumour size as follows: 11/24 (45,8%) had T1 status, 12/24 (50,0%) T2, 1/24 (4.2%) T3 cancer. All were staged N0 and M0. Type I cordectomy was performed in 1/24 (4.2%), type II in 5/24 (20.8%), type IV in 3/24 (12.5%), type V in 12/24 (50%), type VI in 2/24 (8.3%), extended cordectomy in 1/24 (4.2%) patients. In 3/24 (12,5%) patients, primary tumour was localised in anterior commissure. Remaining 21/24 cases had tumours localised on mid-vocal fold.

All of the 24 patients assigned to Group A underwent SL combined with excisional biopsy. Suspicious NBI lesions during the first follow-up visit were visualized on the mucosa (Fig 1) in 14/24 (58.3%) subjects. Nine out of twenty-four (9/24, 37.5%) patients had positive deep margins after the initial TOLM, whereas 1/24 (4.2%) had uncertain deep margin. In 1/24 patient (4.2%) both positive deep margins and positive NBI result were recorded.

The microvascular pattern in NBI and deep margins status were compared with the final histology after SL surgery. The positive final histology in SL (defined as severe dysplasia, cancer *in situ* or invasive cancer) was revealed in 10/24 (41.7%) patients. Among patients with positive histology after SL surgery 10/10 (100%) had suspicious vascular pattern in NBI and 1/10 (10%) had both positive deep margin after the initial TOLM and suspicious vascular pattern in NBI. Out of 10 patients with positive lesion seen in NBI, 2/10 (20%) had positive superficial margin and 2/10 (20%) had close superficial margin.

In Group A, a local recurrence occurred in 1/24 (4.2%) patient 12 months after SL. This particular patient had a T2 tumour and was aged 62. After the initial TOLM, he had both positive lesion in NBI and positive superficial margins.

There was a significant correlation between the suspicious vascular pattern of the mucosa in NBI and the final histology result after SL (Chi2(1) = 12,24; p = 0.00047). There was no significant association between the status of the margins and the final histology result of SL (Chi2(4) = 8.47; p = 0.07571). There was no association between T stage and the vascular pattern of the mucosa in NBI (Chi2() =; p = 0,67732) and between the type of cordectomy and the vascular pattern of the mucosa in NBI (Chi2(), p = 0,55271).

### Group B–A low-risk group according to NBI

To Group B belonged patients with positive or uncertain superficial margins and non-suspicious vascular pattern in NBI. Group B consisted of 52 patients (8 female, 46 male, mean age of 64 and age range of 28–87). Thirty-seven patients (71.2%) had uncertain and 15 (28.8%) positive superficial margins. All patients had non-suspicious vascular pattern of mucosa in NBI. During approximately 23 months follow-up (average 23, range 13–35 months), none of the patients in this group developed recurrence nor presented with suspicious vascular pattern in regular NBI endoscopy (performed every 3–4 weeks).

One patient (1/52, 1.9%) with uncertain superficial margin after initial surgery presented with a suspicious vascular pattern in NBI during one of the follow-up visits, 16 months after the initial surgery. The SL was performed and the final histology confirmed local recurrence.

### Group C–No risk factors group

To Group C belonged patients with negative surgical margins and non-suspicious vascular pattern in NBI. Group C consisted of 51 patients (4 female, 47 male, mean age– 64, age range of 42–90). During approximately 23 months follow-up (range 14–30), none of the patients in the group developed recurrence nor presented with suspicious vascular pattern in regular NBI endoscopy (every 8–10 weeks).

## Discussion

This is the first report confirming a great value of NBI technique in guiding the decision-making regarding follow-up of patients after TOLM in early and moderately advanced glottic cancer. We proved that there is a high correlation between the vascular pattern in NBI and final positive histology after SL. In our cohort, NBI turned out to be a better predictor of final positive histology in SL than positivity of deep surgical margins in the first procedure.

In fact, the prognostic value of surgical margins after TOLM is still under debate. Its prognostic relevance is subjective to sample’s dimensions, sampling technique and experience of a pathologist. Definitions of surgical margins status in laryngeal cancer depend on the T stage of the primary tumour. Some experts distinguish ultra negative margins defined as >1 mm in T1 glottic cancer [10,11] and >3 mm in T2-T3 glottic cancer [10]. In our paper, we decided to unify margins definition criteria for the whole group. Thus, all margins, disregarding the primary tumour T stage (T1-T3), were defined as negative if >1 mm. In the studied cohort, both superficial and deep margins were carefully sampled with intent to preserve a balance between oncological outcome and post-treatment optimal vocal function. Peretti et al. and Crespo et al. reported that the rate of recurrence in cases with positive margins after TOLM ranges from 7% to 37% [12,13]. This is consistent with our study, as the recurrence rate in subjects with positive deep or superficial surgical margins equalled 8% (2/25), whereas the recurrence rate in subjects with positive NBI findings equalled 7% (1/14).

As mentioned above, prognostic value of margin status is still under debate. Some authors claim that positive margins significantly worsen recurrence-free survival (RFS) in patients after TOLM [14,15]. On the other hand, some consider that margins positivity has no impact on RFS. In our study, only 1/24 (4.2%) patient who had both positive margins and suspicious findings in NBI developed recurrence 12 months after SL. What is more, there was no statistically significant correlation between status of margins and the final histological result after SL. In the contrary, the NBI findings correlated strongly with positivity of surgical margins in SL.

For patients with laryngeal cancer treated with TOLM, the management of patients with positive margins in primary surgery is under discussion and there are no clear guidelines for decision making regarding follow-up [16]. On the one hand, European Laryngology Society (ELS) recommends performing immediate second look surgery [17]. On the other hand, some experts claim that watch and wait policy is more appropriate and that it is more important to perform thorough clinical examination instead [18]. Therefore, we aimed in our study to investigate NBI as a tool guiding decision-making regarding follow-up. In our innovative approach only patients with positive deep margin or/and positive lesion in NBI are being qualified to second look surgery. In this study, we showed that patients assigned to Group B benefited the most. Our approach resulted in saving 51 subjects in Group B with positive margins who did not develop recurrence from unnecessary surgical procedure.

The current ELS pathological vessels NBI classification defines suspicious lesions as those having perpendicular vessels in the third dimension pictured as brownish spots. However, majority of authors investigate theses lesions only before TOLM, not after it. Only Lukes et al. showed that after TOLM the majority of recurrences origin from a submucosal growth that is usually whitish in NBI and is covered by a healthy looking mucosa that exhibits very little or no vascular changes. Therefore, to the group of positive lesions in NBI, apart from typical pathological vessels, we also included lesions with amputation of vessels [19].

NBI technique has been recently confirmed to improve the accuracy of intraoperative neoplastic superficial spreading evaluation and accuracy of the tumour resection during TLM in early glottic cancer [20]. Based on our own experience and the results of the study, we proved that the NBI is also a helpful tool in decision-making regarding follow-up in cases with positive margins after TOLM. All patient with suspicious vascular pattern in NBI (10/10 patients) had positive histology (severe dysplasia, cancer in situ or invasive cancer) in SL.

Our study’s main limitation is the time of follow-up (average of 23 months), as some patients may still develop recurrence of cancer later than follow-up period analysed in this study. Furthermore, additional investigation with more subjects is warranted at this time to further validate this technique and change the standard of care.

## Conclusions

Our study provides evidence that NBI imaging may be a useful adjunct to margin status after TOLM and may facilitate clinical decision-making regarding performing the SL in patients with positive or uncertain superficial surgical margins during the first TOLM procedure. However, additional investigation with more subjects is required at this time to further validate the method and to change the standard of care.

## Supporting information

S1 File Minimal data set. (XLSX)
